# The Impact of Acute COVID-19 Infection and Long COVID in Patients with Congenital Heart Disease: A Longitudinal Study by the German National Register for Congenital Heart Disease

**DOI:** 10.3390/jcm15051986

**Published:** 2026-03-05

**Authors:** Cornelia Tremblay, Ulrike M. M. Bauer, Jens Beudt, Stefan Orwat, Gerhard-Paul Diller, Constanze Pfitzer, Paul C. Helm

**Affiliations:** 1 National Register for Congenital Heart Defects, Augustenburger Platz 1, 13353 Berlin, Germany; 2 Competence Network for Congenital Heart Defects, 13353 Berlin, Germany; 3 Adult Congenital and Valvular Heart Disease Center, Department of Cardiology and Angiology, University Hospital Muenster, 48149 Muenster, Germany; 4 Department of Congenital Heart Disease-Pediatric Cardiology, Deutsches Herzzentrum der Charité, 13353 Berlin, Germany; 5 Charité–Universitätsmedizin Berlin, Corporate Member of Freie Universität Berlin and Humboldt-Universität zu Berlin, 13353 Berlin, Germany

**Keywords:** congenital heart defects, CHD, COVID-19, Long COVID, telephone survey, Germany

## Abstract

**Background:** Patients with congenital heart disease (CHD) were considered to belong to a vulnerable group at risk for COVID-19 infection. Our aim was to investigate the severity of acute COVID-19 infection in this patient group as well as the occurrence of sequelae. **Methods:** We performed telephone interviews with all accessible COVID positive CHD patients from our online COVID-19 patient survey. Baseline information was extracted from our nationwide data bank, with further details from hospital discharge letters. **Results:** Ninety-nine patients (or parents) were interviewed (male 50.5%): 28 children, 32 young adults (up to 29 years), and 39 adults (30 years and above). Twenty patients had simple, 38 moderate, and 41 complex CHD (10.1% were cyanotic). In twelve patients the CHD was native, ten underwent univentricular palliation, and the rest had corrective cardiac treatment. Thirty patients had additional non-cardiac risk factors. The acute course of COVID-19 was mild in 50, moderate in 38, and severe in three patients, requiring hospitalization. No deaths occurred. Long COVID symptoms (persisting ≥ 12 weeks) were reported by 31 patients. **Conclusions:** Despite underlying CHD, the severity of the acute course of COVID-19 in our cohort is comparable to that in the general population. Even patients with cyanotic CHD, complex CHD after univentricular palliation, or those with pulmonary hypertension, usually had a mild to moderate course, so that hospitalization was rarely necessary. The percentage of CHD patients reporting Long COVID symptoms (31%) was higher than in the general population. The long-term impact of COVID-19 and Long COVID in CHD patients is unknown and remains to be investigated.

## 1. Introduction

After its first report in December 2019 from Wuhan, COVID-19 (Coronavirus SARS-CoV-2) spread rapidly and quickly developed into a pandemic [1]. Patients with congenital heart disease (CHD) were considered to belong to a vulnerable group at risk [2], but the true risk for this patient group remained unknown. Fortunately, most infected patients made a full recovery, but how many patients in this subgroup were truly affected, how severely, and to what extent Long COVID occurred, was not known.

Several studies now exist on acute COVID-19 infection in CHD patients, most of which focus on hospitalized or more severe cases [3,4,5,6,7]. However, we have only found one article, published by Zhang et al. [8] in April 2025, on post-acute cardiovascular sequelae of COVID-19 in CHD patients. Our study includes patient-reported data and examines the outcomes on the acute COVID-19 infection and the prevalence of Long COVID. To the best of our knowledge, there are no further data on Long COVID in CHD patients available.

From the beginning of the pandemic until the end of 2024, 777,126,421 confirmed COVID-19 cases, and 7,079,925 related deaths have been reported to the WHO [9]. At the same time, 13.64 billion vaccine doses have been administered worldwide [9].

Even though the COVID-19 pandemic is now over, and has developed into a global endemic situation, there are still ongoing waves of COVID-19 infection with newly emerging COVID variants [10,11,12]. The course of the acute infection with current COVID-19 strains is now often less severe, but the cumulative risk of Long COVID increases with each acute COVID-19 infection [13,14]. Undervaccination is associated with an increased risk of severe COVID infection [15], while vaccination against COVID-19 not only reduces the risk of severe cases of illness or hospitalization, but also that of Long COVID [16].

Long COVID is a complex heterogenous multisystemic condition, defined by ongoing symptoms more than 4 weeks after the acute infection [17]. If the symptoms persist or reoccur 12 weeks after the acute infection, lasting at least for two months with no other explanation, it is defined by the WHO as post-COVID-19 [18]. As this study was designed prior to the previous mentioned WHO definition, and we are not able to determine the total length of the Long COVID symptoms with a single patient contact, we continue to use the term Long COVID in this article. The global prevalence has been estimated at 6% of patients who had symptomatic COVID-19-infection [19]. In a review published in 2024 [20], the cumulative global incidence of Long COVID was estimated as 400 million people. Another article by Davies [21] summarizes that most Long COVID cases appeared after a mild acute illness in non-hospitalized patients between 36 and 50 years old, and in about at least 10% of patients following a severe COVID-19 infection.

The list of Long COVID symptoms currently comprises more than 200 symptoms [21]; the most commonly described of which include unremitting fatigue, cognitive impairment, post-exertional malaise, autonomic dysfunction, as well as several other symptoms.

The underlying cause of Long COVID is still not fully understood. There are different hypotheses. One is the persistence of the virus SARS-COVID-19 or its components in the body [22]. Two other hypotheses are autoimmunity or immune compromise with secondary viral reactivation such as herpes virus, Ebstein–Barr-virus, and/or Varicella-Zoster virus [21,22]. Ongoing vascular (endothelial) and/or neuronal inflammation in the body or chronic tissue dysfunction due to organ damage are discussed as well [22]. Other factors such as activation of platelets, microthrombi, reductions in oxygen-usage within the tissue, mitochondrial dysfunction, microbiome dysbiosis, and/or low cortisol levels might have an added effect [21,22].

Almost all studies to date on CHD patients with COVID-19 have only examined the impact of the acute COVID-19 infection [6,23,24,25,26,27,28]. The focus is often on hospitalized patients [24] and/or COVID-19-related mortality, as in a reported cases/controls study [25] from the UK, in which the case-hospitalization and case-fatality rates among more than 86,000 CHD patients (compared to matched controls) were 22.4% (vs. 14.5%) and 6.1% (vs. 3.8%) respectively. Severe CHD complexity, older age, ethnicity, and pulmonary hypertension were associated with worse outcomes. In a retrospective study [26] using a large American inpatient database, CHD patients with COVID-19 were more likely to experience in-hospital mortality and complicated COVID-19 infection. In a large joint North-American and European multicenter review [27] of 1.044 patients, advanced physiological state was more critical to the patient’s outcome than the anatomical complexity of the CHD. The physiologic state [29], as a surrogate of functional impact of the CHD, included the following: NYHA functional class, significant valve disease, ventricular dysfunction, arrhythmia, presence of cyanosis or hemodynamic relevant shunt, pulmonary hypertension, Eisenmenger syndrome, relevant changes in major vessels (dilation or stenosis), and end-organ dysfunction.

The function of the immune system could also play a role, as it is often impaired in CHD patients, as several studies have recently shown [30,31]. A German study by Diller et al. [30] found that an immune deficiency syndrome is more common in patients with congenital heart disease compared to the general population, and Bremer et al. [31] discovered thymic atrophy and secondary immune dysregulation in infants with complex congenital heart disease. Whether this has clinical implications, for example in acute COVID-19 infection or Long COVID, is unknown.

The study by Zhang et al. on COVID-19-related sequelae in children and adolescents [8] found a statistically significant increased risk of various cardiovascular events following COVID-19 infection. Patients with CHD had an increased adjusted relative risk of individual cardiovascular events, such as hypertension, atrial fibrillation, ventricular arrhythmias, myocarditis, heart failure, cardiomyopathy, cardiac arrest, and others. In addition, it was found that immunocompromised individuals and patients who had experienced severe acute COVID-19 infection also had a higher risk. The COVID-19 variant (Delta or Omicron) had no influence on cardiovascular events.

In a metabolic profile study from May 2025 [32], two groups of children infected with COVID-19-acute and post-acute with multisystem inflammatory syndrome were compared with healthy peers. The investigations revealed pathophysiological changes in both affected groups with alterations in the lipid profile, including an increased Apo-B100/Apo-A1 ratio, which is a predictive marker of atherosclerosis. These findings also suggest possible long-term effects on cardiovascular risk.

To gain information on the impact of COVID-19 pandemic in CHD patients and their quality of life, particularly from the patient’s perspective, our group conducted an online COVID-19 patient survey [33] 18 months into the pandemic. Patients were asked about the medical care they received for their CHD during the pandemic, their knowledge of COVID-19, how affected they felt by the pandemic, and whether they had an acute COVID-19 infection, among other things. From the 3655 CHD patients (female 51.9%) who completed the question about the acute COVID-19 infection (3179 patients completed the whole online questionnaire), 4.2% indicated that they have been infected at least once with COVID-19. These formed the basis for our further study, in which we wanted to investigate the acute course of the COVID-19 infection, as well as the long-term effects in this selected patient group.

## 2. Material and Methods

### 2.1. Patient Cohort and Study Design

Our inclusion criteria were the presence of CHD and having had an acute COVID-19 infection. Consequently, we included all the patients from our previous online survey who met these inclusion criteria. Our goal was to perform a telephone interview with all accessible COVID-19-positive patients to get more insights into their course of infection. The ethics approval (EA2/109/21, date: 21 June 2021) applied to both the previous online survey and this subsequent study.

The patients were contacted by phone and asked whether they would like to participate in a telephone questionnaire about their COVID-19 infection. Our questionnaire comprised 20 questions including details about the acute COVID-19 infection, known risk factors, COVID-19 testing, Long COVID symptoms, COVID vaccinations, and medications. The telephone interviews were conducted between October 2021 and May 2022. Since we were unable to reach everyone by telephone in the first round, we sent out emails and letters to maximize the total number of patients, and then called them after receiving their response. Baseline information, such as congenital heart disease, other non-cardiac diagnoses, heart surgeries, and other cardiac intervention, were extracted from our nationwide medical database for CHD patients. Further medical COVID-1-related details were taken from hospital or rehabilitation discharge letters.

The patients were grouped by age into three groups: children (0 to 17 years), young adults (18 up to 29 years), and adults (30 years and older). For each patient, the following data were collected: underlying CHD, other cardiac diagnosis, heart surgeries, cardiac interventions, medical treatment (including thromboprophylaxis), genetic syndromes, non-cardiac risk factors (immunocompromise, pulmonary disease, obesity, diabetes, smoking, hypertension, pregnancy, cancer), and comorbidities, onset of the acute COVID-19 infection, performed COVID-19 test, possible source of infection, as well as symptoms, severity, and duration of the acute COVID-19 infection, need for hospitalization, treatment, complications, ongoing symptoms after the curation and their duration, Long COVID symptoms (defined as three months after the acute infection), rehabilitation or any other treatment for Long COVID, and COVID-19 vaccinations.

Congenital heart defects were classified as simple, moderate, and complex according to the Warnes criteria [34]. Depending on the course of the acute COVID-19 infection and severity of the illness, patients were classified into the following categories: asymptomatic, mild, moderate, and severe. We took into account the acute symptoms, the duration of the illness, the occurrence of fever, bedriddeness, and the need for hospitalization. Patients with mild cold symptoms were classified as mild. Persistent fever, more flu-like symptoms and “bedriddeness for a week and more” were criteria for a moderate course, “hospital admission related to COVID-19” with more advanced treatment for a severe course.

### 2.2. National Register for Congenital Heart Defects

The National Register for Congenital Heart Defects [35] was founded in 2003 with the goal to collect nationwide data of patients with CHD. By April 2025, around 60,000 patients had voluntarily participated and been registered. At the outset, the medical data of each CHD patient is recorded anonymously. The baseline and follow-up data forms the basis for various research projects, including longitudinal studies focusing on disease progression, surgical outcomes, medical care, and quality of life.

### 2.3. Statistical Analyses

Data were entered in a study-specific data bank and analyzed with SSPS, Version 25. Categorical variables are represented as numbers and percentages. We performed a chi-squared test to analyze whether the acute course of COVID-19 and non-cardiac risk factors influenced the risk for Long COVID.

## 3. Results

### 3.1. Study Cohort and Patients Characteristics

From 152 COVID-19-positive patients extracted from the former online survey, 99 participated in the telephone interview (65.1%), two patients were not interested, and the remaining we were unable to contact. The sex distribution was balanced (male 50.5%). The age distribution was as follows: 28,3% children, or adolescents, 32.3% young adults, and 39% adults (for further details see Table 1). Only one patient was older than 60 years.

### 3.2. Medical and Disease-Related Patient Data

**Figure 1:**
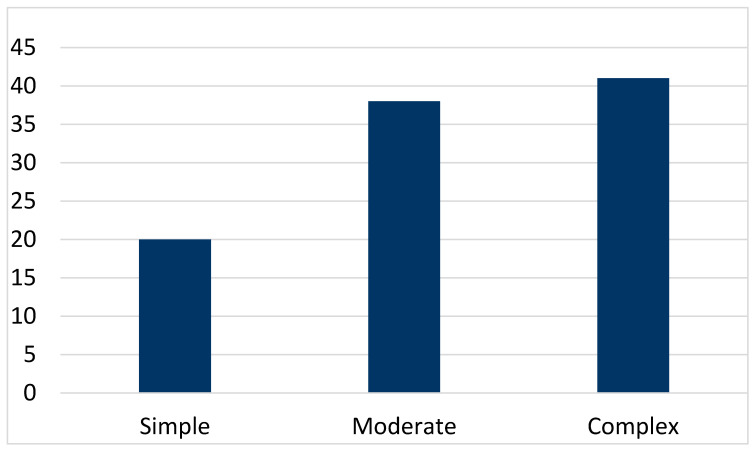
Distribution of congenital heart disease (CHD).

In our patient cohort, 80% had either a moderate or complex congenital heart defect (for further details see Figure 1). Most patients had normal oxygen saturation, and only a small group (10.1%) was cyanotic.

The group of patients with native congenital heart defects included 12 patients. Four of these had more complex lesions: Ebstein’s anomaly (n = 2), congenital corrected transposition of the great arteries (ccTGA, n = 1), and double inlet left ventricle/pulmonary stenosis (DILV/PS, n = 1). The remaining 87 patients have undergone either corrective or palliative surgery, and/or another form of cardiac intervention. Of these, ten patients underwent univentricular palliation: Glenn surgery (n = 1), Fontan-operation (n = 8), and Kawashima procedure (n = 1).

The following patients had CHD-associated risk factors: Three patients had pulmonary arterial hypertension, one of whom required additional oxygen as baseline therapy, while the two others were treated with medical therapy only. Heart failure was present in two and cyanosis in ten patients. One patient had right atrial isomerism with asplenia.

In addition to their congenital heart defect, 30 patients reported having at least one of the following non-cardiac risk factors: immunocompromise (n = 2), arterial hypertension (n = 12), diabetes mellitus (n = 3), obesity (n = 18), bronchial asthma/chronic lung disease (n = 8), and nicotine abuse (n= 5). Genetic anomalies were present in 13 patients: Trisomy 21 (n = 7), Noonan Syndrome (n = 1), Holt–Oram Syndrome (n = 1), Di-George Syndrome (n = 3, one of these diagnosed with T-cell-deficiency), Microdeletion 17 (n = 1). One patient had an absent left kidney. Two patients had received cancer treatment in the past, one of whom was still undergoing long-term hormone-modulating treatment. One patient had suffered a stroke. Another patient tested positive for COVID-19 during her pregnancy (at 25 weeks). Fortunately, the further course of her pregnancy was uneventful, and she delivered a healthy, full-term newborn.

The majority of our patients (n = 98) were unvaccinated at the time of the acute COVID-19 infection. Fifty-eight patients first developed COVID-19 symptoms, and 41 patients tested positive while they were still asymptomatic. Twenty-two patients underwent a rapid test, only five performed the test by themselves, and the majority (92.9%) were diagnosed by PCR-Test. In a minority, the rapid test was followed by a PCR-Test.

In more than two-thirds of cases (70.7%), another family member or close contact (often a housemate) was also infected.

**Figure 2:**
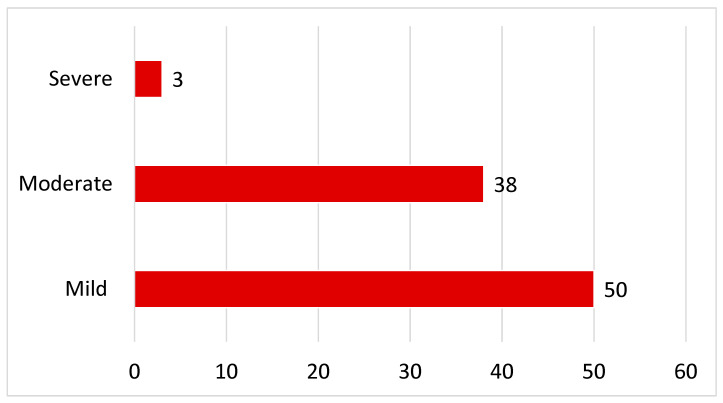
Course of the acute COVID-19 infection.

The majority of patients (97%) did not need to be hospitalized. Eight patients were completely asymptomatic and learned of their COVID-19 infection through a positive test performed due to COVID-19 contact. Fifty patients had a mild, rather paucisymptomatic course with typically described mild cold symptoms. Thirty-eight patients were multisymptomatic with flu-like symptoms, high fever for several days, prolonged bed rest, severe malaise, etc., and therefore categorized as having a moderate course. Three patients had to be admitted to the hospital; out of these, one was admitted to the intensive care unit (see Figure 2, and for details see Table 2). Further support, such as dialysis and ECMO, was not required in any patient; no deaths occurred.

**Figure 3:**
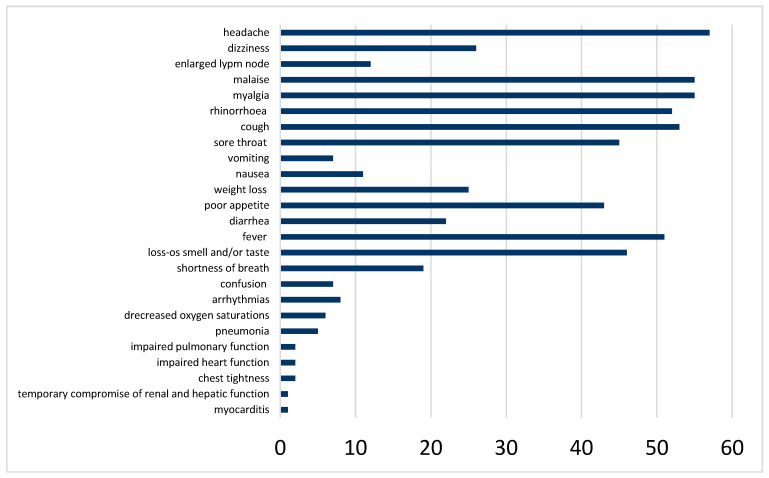
Prevalence of reported acute COVID-19 symptoms.

Over half of the patients reported fever and/or cold-associated symptoms such as runny nose, sore throat, and cough (detailed listing of the symptoms in Figure 3). Flu-like symptoms, such as malaise, muscle and/or joint pains were present in 55.6% of the patients. More severe respiratory symptoms like shortness of breath were reported by 19 patients, six patients had decreased oxygen saturations, two impaired pulmonary function, and five had pneumonia. The percutaneous oxygen saturation was often not measured because the symptoms were only mild. Gastrointestinal symptoms were reported by 52 patients. From the cardiological perspective, two patients reported chest tightness, eight reported new onset or worsening of arrhythmias, two reported impaired cardiac function, and one patient developed concomitant myocarditis. One patient reported a temporary worsening of a preexisting impaired renal function and protein deficiency due to temporary impairment of the liver function. Other severe complications such as stroke, ARDS, dilated coronary arteries, PIMS, acute renal or hepatic failure, and/ or thromboembolism, did not occur in our cohort.

Two patients received steroids and four antibiotic treatments. Virostatic therapy or monoclonal antibodies were not administered in any patient of our cohort. Treatment with oxygen was necessary in two cases, both were admitted to the hospital.

The average time to recover was 12 days; six in children, 12 in young adults, and 16 in adults. The range was between one day and up to seven weeks. The severity of the underlying congenital heart defect did not have an impact on the recovery time (simple CHD 11 days, moderate CHD 14 days, complex CHD 12 days).

The majority of patients had one course of an acute COVID-19-infection (n = 86), 12 patients had it twice, and one patient was infected three times. In seven patients the second course of COVID-19 was milder than the first infection, two reported similar symptoms, and in three the subsequent disease was even more severe.

Following the acute phase of the COVID-19 infection, thirty-six patients reported several short-lasting symptoms in the first weeks after the acute infection, which disappeared shortly thereafter without any treatment. A third of the patients (n = 33) complained of fatigue, eighteen mentioned temporary concentration and memory problems, and 16 had trouble finding the right words.

**Figure 4:**
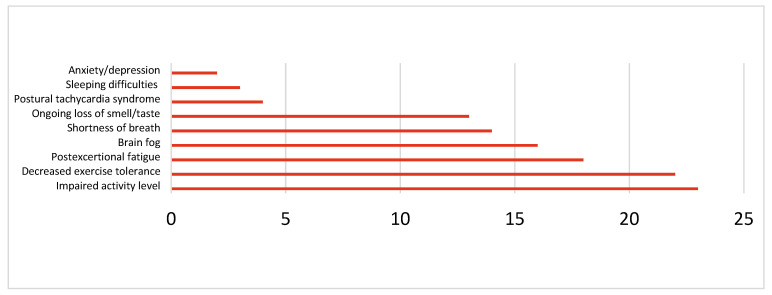
Long COVID symptoms and their prevalence.

In total, thirty-one patients (31.3%) described experiencing Long COVID symptoms (ongoing symptoms after 12 weeks). Eighteen were female and 13 were male. The age distribution was as follows: two were children, nine young adults, and 20 adults. The reported symptoms with their prevalence are listed in Figure 4.

The two affected children (2 out of 28, 7%) were a 7-year-old girl and 11-year-old boy; their described symptoms were impaired activity level, decreased exercise tolerance, and shortness of breath. For the adult population, the occurrence of Long COVID symptoms was more frequent in older (51%) than in younger adults (28%). The oldest patient in our cohort (mid 70s), with a native double inlet left ventricle, associated pulmonary stenosis (baseline oxygen saturation 97%), and a pacemaker, suffered from a mild acute COVID-19 infection, but subsequently struggled with Long COVID symptoms.

Only three patients underwent rehabilitation for Long COVID (details see Table 3).

**Figure 5:**
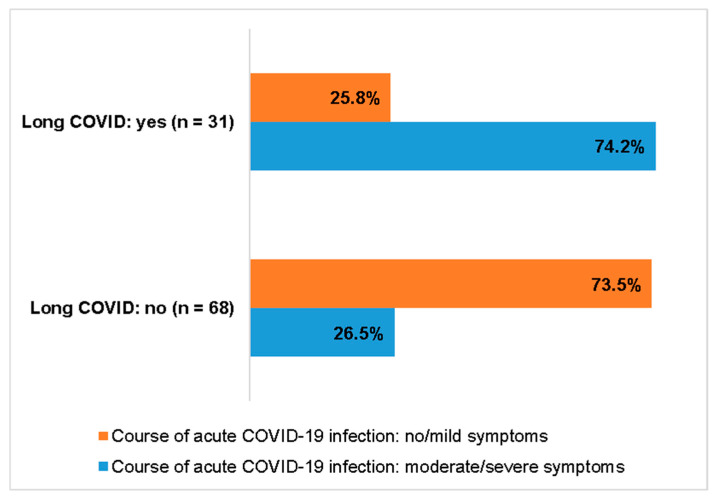
Severity of acute COVID-19 infection and Long COVID.

**Figure 6:**
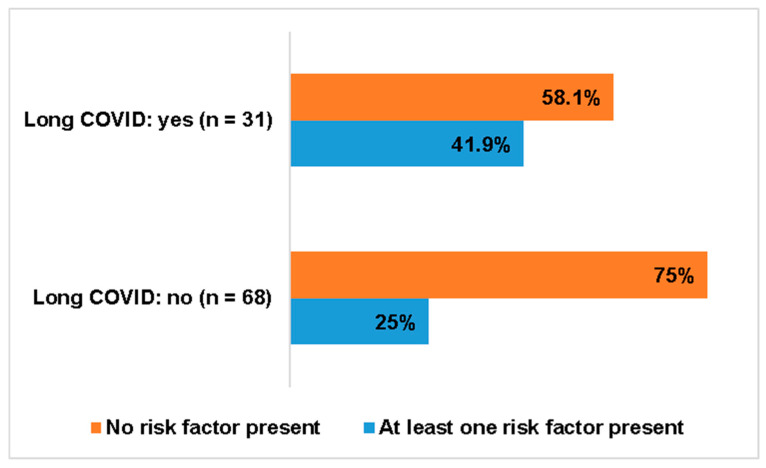
Non-cardiac risk factors and Long COVID.

Comparing the two groups of patients with and without Long COVID, there were remarkable differences (31 versus 68). In the group of Long COVID there were significant more patients with a moderate or severe acute course of COVID-19 (see Figure 5, Chi-squared test: *p* < 0.001). In terms of non-cardiac risk factors, there was also a higher tendency to develop Long COVID when at least one non-cardiac risk factor was present (see Figure 6, Chi-squared test: *p* = 0.089). For instance, one of the immunocompromised patients with microdeletion 22q11 and a t-cell defect, on weekly antibody treatment, developed Long COVID after a moderate course of her acute COVID-19 infection.

Of the 12 patients who have been infected with COVID-19 twice, three developed Long COVID symptoms. All other Long COVID patients had only one preceding infection. The patient who had COVID-19 three times did not develop any long-lasting symptoms.

As part of their long-term medication, twelve patients received oral anticoagulant therapy with phenoprocoumon, nine were on treatment with ASA, and one patient had Apixaban.

The majority of patients taking vitamin K antagonists performed self-monitoring. During the acute COVID-19 phase, seven patients reported INR-levels within the therapeutic range, three had to reduce the dose due to an increase in INR, one increased the dose due to a low INR, and one patient reported that the INR was simply not stable.

With regard to the vaccinations, only one patient reported having been vaccinated before the COVID-19 infection. Seventy-nine patients were vaccinated after their COVID-19 infection; of these, 30 patients received (by the time of the telephone interview) one dose, 35 had received a booster vaccination, and 14 received in total three vaccinations. Nineteen patients were not vaccinated at all. Ten out of these were too young, one patient had a vaccination appointment scheduled, two deferred it, as their antibodies were still high, and six patients were either unsure about the vaccination or decided against it.

## 4. Discussion

In comparison to other studies on patients with CHD and acute COVID-19 infection, we explored the patients’ perspectives, which was particularly important to obtain more information about Long COVID.

Firstly, we wanted to investigate how the COVID-19-positive CHD patients experienced their acute COVID-19 infection in terms of symptoms and severity. In addition, we were interested in analyzing the occurrence of Long COVID symptoms in this patient group, as it can add another layer of complexity in the future treatment in these already challenging patients.

Published data on COVID-19-related morbidity and mortality in CHD patients vary due to other study compositions, and results are only partially comparable as they relate to a different research focus and point in time during the pandemic, for example unvaccinated inpatients [6] versus vaccinated patients [5]. In our cohort with mostly unvaccinated patients (98%), the severity of the acute course of the COVID-19 infection is comparable to that of the general population. Even challenging patients with a cyanotic CHD, a complex heart lesion who underwent univentricular palliation, or those with pulmonary hypertension, had only mild to moderate COVID-19 course and, in most cases, hospitalization was not necessary. In terms of acute symptoms, our results are similar to Broberg’s large multicenter review with ten times as many CHD patients, e.g., the proportion of asymptomatic patients (8% vs. 6%), as well as occurrence of fever (55% vs. 44%), both less frequent than in the general population [27]. In a European study by Schwerzmann et al. [28] with almost the same number of CHD patients (n = 105), including mainly adults, the percentage of patients with a complicated course was much higher compared to our study, including five deaths. A correlation between the higher average age of this patient group with likely more comorbidities and an increased risk of a more complicated course of COVID-19 is conceivable and comparable to the general population. Their most important predictors for a complicated course were age, obesity, and various other comorbidities, and specific to the heart, an unrepaired cyanotic CHD or Eisenmenger Syndrome. Another study by Bradley et al. [29] stated in addition to cyanosis, an impaired physiological stage, arrhythmia, and pulmonary hypertension as major risk factors for a severe course of COVID-19 infection in CHD patients.

Sabatiano et al. [36] revealed that CHD patients in general had a less severe COVID-19 course than expected. The physiological state appears to be relevant for the severity of acute COVID-19 infections as many other influencing factors, which also apply to the general population, such as age, comorbidities, risk factors, etc. [28,29]. A British case-control study [5] with 318,135 health records revealed a markedly reduced COVID-19-related hospitalization (0.5% vs. 15.8%) and mortality rates (0.5% vs. 4.6%) in vaccinated CHD patients compared to unvaccinated peers. However, in comparison to vaccinated controls, the vaccinated CHD patients still had an increased risk of hospitalization and death. Due to the relatively small number of patients in most of the studies, including ours, definitive conclusions about the true risk of COVID-19 in CHD patients remain limited. In a 2020 article by Radke et al. [2], an attempt was made to categorize risk groups for different CHD patients. Taking into account the knowledge now gained, CHD patient groups should be reevaluated in terms of their individual true risk. This can provide clarity for patients and help treating physicians to identify which CHD patients are actually at risk, should be monitored more closely, and would benefit from early hospital admission in the event of future pandemics. It could also help to alleviate fears of this patient group [37], and in future, isolating measures could be minimized, without putting truly vulnerable patients at risk.

Meanwhile the perception from the public towards Long COVID changed dramatically and it is now recognized as a separate disease. Significant efforts have been made to understand this relatively new disease, which involves multiple systems and can have devastating effects on all organ systems and may cause lifelong consequences.

In a Lancet paper [38], the incidence of Long COVID in unvaccinated not hospitalized patients ranged between 10 and 35%, for unvaccinated hospitalized between 50 and 85% and for vaccinated individuals between 8 and 12%. In our cohort, the percentage of patients reporting Long COVID symptoms (31%) was at the higher end, with an incidence increasing towards the older age group. The average age of our cohort is younger compared to the general population due to a lower life expectancy, particularly among patients with moderate or severe congenital heart disease, and to the nature of the initial study, which used an online questionnaire, making it more likely that younger patients participated.

In our data, there was no clear preference regarding gender, but there was a significant correlation between moderate or severe acute COVID-19 infection and the likelihood of developing Long COVID. The presence of at least one non-cardiac risk factor also revealed a tendency towards developing Long COVID as well. Our findings were consistent with many of the following general risk factors for Long COVID listed in a recent review article [29], such as pre-existing conditions, female, deprived initial illness, multisymptomatic, more sick in the initial COVID-19 illness, unvaccinated or under-vaccinated, was unable to rest during the initial illness, no antivirals given during initial illness, and gets recurrent COVID-19 infections.

The wide ranges of incidence reveal one of the major challenges in the research on Long COVID. Some studies took place at an early stage of the pandemic, others at a later stage, which led to a different vaccination status in many patients and the illness being caused by different COVID-19 variants. In addition, different groups (in terms of non-hospitalized/hospitalized, age, comorbidities) were studied with different research questions, and even the definition of Long COVID as an inclusion criterion varied widely. A comparison of these data is therefore only possible to a very limited extent.

A state of the art review in the European Heart Journal on the current state of research on Long COVID, with a focus on the cardiovascular system, highlights the known effects of COVID-19 on the heart and presents various cardiac examinations as part of post-COVID investigations [39]. These cardiovascular findings, such as myocarditis, right ventricular injury, myocardial infarction, vasculitis, thrombosis, and POTS, as well as their frequency, vary widely between the different mentioned studies, which is partly related to different study design. Another challenge is that the underlying pathogenesis is not yet fully understood; following possible mechanisms are discussed: cytotoxic injury, dysregulation of renin–angiotensin–aldosterone system, endothelitis, thrombo-inflammation, and dysregulated immune system. The long-term effects of COVID-19 on cardiovascular risk are also not completely known. The study by Zhang et al. [8] to investigate COVID-19-related sequelae in children and adolescents revealed a statistically significant increased risk of various cardiovascular events after COVID-19 infection, including children with CHD with an increased adjusted relative risk of individual cardiovascular outcomes. Another recently published metabolic profiling study showed an increased cardiovascular risk [32] in children without a congenital heart disease after COVID-19 infection. These latter findings could be particularly relevant for patients with congenital heart disease, as they already have an increased cardiovascular burden at the outset.

Since CHD patients were not specifically considered in most Long COVID studies, the long-term prognosis regarding the effects of acute COVID-19 infection and Long COVID on their cardiovascular health is unknown. Therefore, our patients, especially those who had moderate to severe acute COVID-19 infection and are suspected of having Long COVID, should be thoroughly examined (e.g., CMR) to determine the presence of COVID-19-related cardiac changes and to understand their potential impact on an often already compromised cardiovascular system.

Overall vigilance to better monitor patients for Long COVID symptoms has improved in recent years but needs to become an integrated part of routine cardiac follow-up. Since the routine imaging modality is echocardiography and a CMR study [40] in healthy athletes revealed cardiac changes despite an echocardiography showing no abnormal findings, advanced cardiac imaging methods should be considered. A more comprehensive cardiovascular examination as part of a research study could help to understand the full extent of the COVID-19-virus-related impact on the cardiovascular system in CHD patients and subsequently tailor further care and cardiac therapy to each individual. Furthermore, additional research is needed to fully understand the underlying pathophysiology of Long COVID and hopefully find a treatment. Till today, causal therapy for Long COVID does not exist and rehabilitation offers are limited.

Last but not least, the additional burden on society with an estimated annual global economic impact of 1 trillion US Dollar [20] and on the healthcare system due to COVID-19-related long-term chronification remains an additional major challenge.

### Limitations

As is often the case in the field of congenital heart disease, the total number of patients is relatively small and the underlying CHDs are very heterogeneous, which makes statistical analyses difficult. Patients who died as a result of COVID-19 infection are not included in this study. Our data are possibly skewed towards patients who complete online questionnaires. We might also have a certain bias, for e.g., recall bias, as the data are self-reported. In addition, not all elderly patients are capable to use the computer nor have access to the internet and will therefore be excluded. As our cohort consisted of unvaccinated patients, the generalizability of our findings to vaccinated and older patients is limited. Due to the study design, clinical assessment of Long COVID was not possible. For all these reasons, our conclusions are limited.

## 5. Conclusions

The course of the acute COVID-19 infection in our cohort, despite an underlying CHD, appears to be comparable to the general population. The severity of the acute infection also tended toward a mild to moderate course, even in patients with complex CHD, cyanosis or who underwent univentricular palliation, as well as those with pulmonary hypertension. A closer examination of this patient group with much larger patient samples and a focus on the physiologic state could help to define CHD patients who are really at risk.

Long COVID in itself is not yet fully understood and the impact on patients with CHD even less so. In contrast, the percentage of patients with Long COVID appears higher in our cohort compared to the general population at that time. According to our best knowledge there is only one other study about Long COVID in CHD patients and the questions of whether CHD patients have an increased risk of developing Long COVID at baseline and with additional non-cardiac risk factors remains. Therefore, we need further studies in CHD patients to gain a better understanding of Long COVID in this patient group and its secondary effects on the different organs, especially the cardiovascular system. A longitudinal study in CHD patients after COVID-19 infection to monitor Long COVID symptoms and assess the long-term impact on the cardiovascular system would provide clarity and help to understand the specific new challenges that will arise. Even a follow-up on our cohort could also be helpful in gaining more insights into the further course of Long COVID in our CHD patients.

A big question for the future remains: how severe is the organ damage in CHD patients caused by acute COVID-19 infections as well as Long COVID, and are there additive effects to the baseline changes, especially in the cardiovascular system, which could lead to increased morbidity (e.g., cardiovascular events) and mortality in this patient group in the following years. Is there a need to adjust future care and treatment?

## Figures and Tables

**Table 1:** Patients’ demographics and COVID-19 disease.

		Total	Children	Middle Age	Old
		1–76 Years	1–17 Years	18–29 Years	30–76 Years
		n = 99	n = 28	n = 32	n = 39
Age in years (Mean ± Standard Deviation)		26.4 ± 15.7	8.7 ± 4.6	22.4 ± 3.2	42.4 ± 10.2
Gender (female)		49.5%	46.4%	43.8%	56.4%
CHD severity	simple	20.2%	14.3%	40.6%	7.7%
	moderate	38.4%	46.4%	28.1%	41%
	complex	41.4%	39.3%	31.3%	51.3%
Risk factors (cardiac and non-cardiac)		39.4%	10.7%	46.9%	53.8%
Immune deficiency		2%	3.6%	0%	2.6%
Chromosomal anomaly		13.1%	28.6%	9.4%	5.1%
Acute COVID-19	asymptomatic	8.1%	14.3%	9.4%	2.6%
	mild	50.5%	60.7%	53.1%	41%
	moderate	38.4%	21.4%	37.5%	51.3%
	severe	3%	3.6%	0%	5.1%
Long COVID		31.3%	7.1%	28.1%	51.3%

**Table 2:** Three patients with a severe course of acute COVID-19 infection.

	Patient	CHD	Risk Factors	COVID Diagnosis	COVID Therapy
1.	52 y, female	VSD (closed)	Bronchial asthma	Pneumonia	Inpatient for 9 days: oxygen and antibiotics. Rehabilitation: 36 days.
2.	7 y, female	ASD (closed)	Primary ciliary dyskinesia, bronchial asthma, microdeletion 22q11 with T-cell-defect	Acute COVID infection with impaired general condition Long COVID (ongoing dyspnea)	Inpatient for 5 days: antibiotics, steroids, anticoagulation (Factor Xa inhibitor), fluids (no supplementary oxygen was required).
3.	30 y, male	AVSD (repaired)	Trisomy 21, obesity	Severe bilateral COVID-19 pneumonia with secondary respiratory failure, concomitant myocarditis with arrhythmia, severe vitamin D deficiency	Inpatient for 26 days (including ICU): oxygen, mechanical ventilation (13 days).

y = years, ASD = Atrial Septal Defect, AVSD = Atrioventricular Septal Defect, VSD = Ventricular Septal Defect, ICU = Intensive Care Unit.

**Table 3:** Three patients, who underwent rehabilitation for Long COVID.

	Patient	CHD	Risk Factors	Long COVID Symptoms
1.	52 y, female	VSD (repaired)	Bronchial asthma	Reduced lung capacity
2.	49 y, male	SVD (repaired), pacemaker	Compensated renal insufficiency	Decreased exercise tolerance, shortness of breath, Fatigue-Syndrome, and ongoing muscle pain
3.	55 y, female	AS, status post artificial valve replacement	Bronchial asthma, small fiber neuropathy	Shortness of breath, Fatigue-Syndrome, brain fog, and ongoing sleeping difficulties

y = years, VSD = Ventricle Septum Defect, SVD = Sinus Venosus Defect, AS = Aortic Stenosis.

## Data Availability

Data cannot be shared for data protection reasons.
